# Alcohol use disorder and associated factors among human immunodeficiency virus infected patients attending antiretroviral therapy clinic at Bishoftu General Hospital, Oromiya region, Ethiopia

**DOI:** 10.1371/journal.pone.0189312

**Published:** 2018-03-06

**Authors:** Jemal Abdella Bultum, Niguse Yigzaw, Wubit Demeke, Mekuriaw Alemayehu

**Affiliations:** 1 Bishoftu General Hospital, Bishoftu, Oromia region, Ethiopia; 2 Department of Psychiatry, School of Medicine, College of Medicine and Health Sciences University of Gondar, Gondar, Ethiopia; 3 Out Patient Department, Amanuel Mental Specialized Hospital, Addis Ababa, Ethiopia; 4 The Institute of Public Health, College of Medicine and Health Sciences, University of Gondar, Gondar, Ethiopia; National Institute of Health, ITALY

## Abstract

**Background:**

Alcohol consumption among patients with HIV/AIDS increases the burden of the disease. HIV/AIDS is an epidemic among Sub-Saharan African countries. Excessive use of alcohol causes a large degree of health problems, social and economic burden in societies. However, the prevalence and associated factors of alcohol use disorder among this group of people has not been studied very well. Therefore, this study sought to assess the magnitude and associated factors of alcohol use disorder among HIV patients attending the antiretroviral (ART) clinic.

**Methods:**

A hospital based cross sectional study design was conducted at Bishoftu General Hospital from May to June 2015. Systematic random sampling technique was used to select the study participants. Data was collected by face to face interview and chart review. Alcohol Use Disorder Identification Test (AUDIT) was used to assess alcohol use disorder. Bivariate and multivariate logistic regression analysis was carried out to identify associated factors and P-value < 0.05 was taken as statistically significant.

**Results:**

A total of 527 participants were enrolled in the study with a response rate of 100%. The prevalence of alcohol use disorder (AUD) was 14.2%. Factors associated with alcohol use disorder were educational status AOR = 8.5 (95%CI: 1.70, 42.99), social support AOR = 0.5(95%CI: 0.26, 0.95), cigarette smoking AOR = 3.49(95%CI: 1.01, 12.13), khat chewing AOR = 5.11 (95% CI: 1.60, 16.33), family history of alcohol use AOR = 3.58 (95% CI: 1.52, 8.47), and missing ART drugs AOR 3.05 (95% CI: 1.302, 7.131).

**Conclusion:**

The prevalence of alcohol use disorder was high as compared to similar epidemiological studies. Educational status, social support, cigarette smoking, khat chewing, and family history of alcohol use were independent predictors. Providing health education about alcohol use and proper screening of alcohol use disorder among patients with HIV/AIDS is crucial. Strengthening the referral linkage with the psychiatric unit will decrease the burdens of the problem.

## Introduction

Alcohol is a psychoactive substance with dependence producing properties that has been widely used in many cultures for centuries. Harmful use of alcohol causes a large degree of health problem as well as social and economic burden in societies [1–3].

Excessive use of alcohol causes or elevates the risk for alcohol-related problems or complicates the management of other health problems leading to what is known as alcohol use disorder. According to epidemiologic research, men who drink more than 4 standard drinks in a day (or more than 14 per week) and women who drink more than 3 in a day (or more than 7 per week) are at increased risk for alcohol-related problems[4, 5]. The lifetime risk for serious and repetitive alcohol problems is almost 20% for men and 10% for women, regardless of a person’s education or income; the alcohol use disorder is known to decrease life span by about 10 years[5].

Alcohol use is common among HIV-infected persons, with data from national samples indicating that 50% of those who reported any alcohol use are associated with worse HIV treatment outcomes [6, 7]. Patients with hazardous levels of use are neither to receive antiretroviral therapy nor to be adherent to therapy and achieve virology suppression. Screening, intervention and referral to care for alcohol use disorder is an integral part of clinical care for individuals with HIV infection [6–10].

Even an intermittent use of alcohol can complicate the clinical management of HIV/AIDS patients by worsening the outcomes of treatment and condition of patients, diminishing adherence to medications, increasing risk the of hepatic injury, reducing the ability to practice safer sex, increasing the risk of side effects of medication, and changing the pharmacokinetics of prescribed drugs[11]

The prevalence rate of alcohol use in the HIV- infected population is higher compared to HIV- negative population. Studies show that rates of heavy drinking in HIV-infected population is at least twice that of the non-HIV-infected population. Alcohol use among HIV patients plays an important role in their health outcomes and complicates the infection process, contributing to co-morbid diseases and drug interactions [9–11].

Although mortality rates among HIV-infected people have declined with the advent of combinations antiretroviral therapy (ART), patients with substance use disorders have benefited less from the treatment [12].

Alcohol consumption adds fuel to the HIV epidemic in Sub-Saharan Africa (SSA) countries. SSA has the highest prevalence of HIV infection and heavy epidemic drinking in the World. The impact of alcohol consumption on the burden of disease and injury is large in low income countries, with a relatively high consumption in Sub-Saharan Africa and South America, where on average, 30% of all the alcohol-attributable burden is due to infectious diseases. In Sub Saharan African countries, particularly in Nigeria, infectious diseases make up 50% of the overall alcohol-attributable disease burden [13, 14].

Substance misuse is a growing problem in developing countries. Like any other SSA countries, the prevalence of hazardous and dependent alcohol use disorders among HIV patients in Ethiopia is also said to be high. Furthermore, in our review of Ethiopia we found a widespread use of khat and alcohol in the general population. For instance, a recent study at Jimma Referral Hospital revealed that the overall prevalence of Alcohol Use Disorder (AUD) among HIV/AIDS patients, attending the ART Clinic was 32.6%, AUD was higher among patients who did not take their ART medication as prescribed compared to patients who were taking their ART drugs appropriately [1, 15, 16]. Therefore, this study was designed to assess the magnitude and associated factors of alcohol use disorder among HIV patients on ART follow up at Bishoftu General Hospital, Oromia Region, Ethiopia. The finding could provide useful information to policy makers for designing proper interventions.

## Method

### Study design

A facility based cross-sectional study was carried out to assess the magnitude and associated factors of alcohol use disorder among HIV patients on ART follow up at Bishoftu General Hospital, Oromia Region, Ethiopia.

### Study settings and population

The study was conducted at Bishoftu General Hospital, located in East Showa Zone, Oromia Regional State. The hospital catchment population is about one million (480,000 male and 520,000 female). The hospital has an ART unit where 251 HIV positive pregnant women were receiving ART to reduce the risk of mother to child transmission. In the clinic, testing and counseling services were given to 45,494 adults and children within a year and 54.2% of these received ART. ART survival rates during the12 months were 82%. On average, the daily flow of patients to the ART follow up clinic was about 50 patients.

The sample size (n) was computed by single population proportion formula n = [(zα/2)^2^ x P (1-P)]/d^2^, by assuming a 95% confidence level of Z_α/2_ = 1.96, margin of error 4%, and proportion of AUD among PLWHA to be 32.6% according to a study in Jimma [17]. By considering this, the calculated sample size was 527. The participants of the study were over 18 years of age HIV/AIDS Victims who visited the ART follow up clinic of Bishoftu General Hospital during the data collection period.

The systematic random sampling technique was used to select the participants. We used the monthly patient flow to calculate the participant selection interval (k = 2), and every other two participants were selected, and severely ill participants were excluded. The study period was from 15^th^ May to 15^th^ June, 2015.

### Data collection instrument and procedures

AUDIT is a 10-items alcohol screening instrument which emphasizes the identification of alcohol use disorder for the last 12 months [18]. It is developed by the World Health Organization in 1982 and has been found most effective in identifying subjects with less severe drinking problems, such as hazardous drinking, harmful drinking, and alcohol dependence (sensitivity, 94.1%, specificity, 91.7%). The instrument is very important to identify problematic alcohol use at an early stage as well as AUD[19]. The Oslo-3 Social Support Scale was used to measure social support and HIV Stigma Scale and Subscales was used to measure perceived stigma.

The data collection instruments such as AUDIT and Oslo-3 social support scale were pre-tested at the Black Lion Hospital on 5% of the sample. The instruments were prepared in English and translated to Oromifa (local language) and again back to English to confirm the correctness of the translation. Nurses, psychiatry professionals and supervisor were recruited to collect the data. A day training was given to the data collectors and supervisors on the data collection tool and sampling techniques. Supervision was held regularly during data collection. The collected data was reviewed and checked for completeness and relevance on each day of the data collection period.

### Definition of variables

The dependent variable was alcohol use disorder, and the independent variables were socio-demographic variables, like family history, history of substance misuse, situational factors, and environmental factors. The variables were defined as categorical variables as follows:-

**Social Drinker**: the total AUDIT scores of the drinkers among 1 to 7.
**Hazardous Drinking**: the total AUDIT scores of the drinkers among 8 to 15.
**Harmful Drinking**: the total AUDIT scores of the drinkers among 16 to 19.
**Alcohol Dependence**: the total AUDIT scores of the drinkers among 20 to 40.
**Alcohol use disorder**: a person who scored AUDIT scores ≥ 8.
**Oslo-3 Social Support Scale (OSS-3)** those score <8 was classified as poor support (27)
**HIV Stigma Scale and Subscales** those who score ≥ 80 has perceived stigma
**Missing ART**: individuals who missed one or more days of ART drugs.

### Data processing and analysis

Data was checked and coded for its completeness and entered into Statistical Package for Social Sciences (SPSS) version 20. Both descriptive and inferential statistics procedures were undertaken. Tables and figures were used to present the data. Both bivariate and multivariate binary logistic regression model was used to identify factors associated with alcohol use disorder. Crude and adjusted odds ratios with 95% confidence interval were used to determine the strength of association between dependent and independent variables. Variables with P-value≤0.05 were considered as significant.

## Results

A total of 527participants were enrolled with a response rate of 100%. Most of the participants, 313 (59.4%), were female. Four hundred forty- four of them (84.3%) were urban residents. The mean (±SD) age of the respondents was 36.54 (± 9.49) years.

Two hundred seventy- four (52%) were Oromo by ethnicity,184 (34.9%) Amhara, and 32 (6.1%) Gurage. About eighty percent (79.5%; n = 419) were Orthodox Christians, followed by 89(16.9%) Protestants. Two hundred ninety-four (55.8%) of the participants were married and 103(19.5%) single. The educational status of 288 (47.1%) of the respondents was found to be in the category of primary school, followed by 24.9% secondary level. About 54.5%of the respondents were employed. (Table 1)

**Table 1:** Socio-demographic and clinical variables of AUD among people living with HIV were attending at Bishoftu General Hospital in 2015 (n = 527).

Variables	Frequency, n (%)
**Sex**	
Male	214(40.6)
Female	313(59.4)
**Age**	
18–24	37 (7)
25–34	199 (37.8)
35–44	187 (35.5)
45–54	76 (14.4)
55 and above	28 (5.3)
**Ethnicity**	
Oromo	274 (52)
Amhara	184 (34.9)
Gurage	32 (6.1)
Tigre	29 (5.5)
Other	8 (1.5)
**Religion**	
Orthodox	419(79.5)
Protestant	89(16.9)
Muslim	16(3)
Catholic	3(0.6)
**Education**	
Unable to read & write	96 (18.2)
Primary (1–8)	248 (47.1)
Secondary (9–12)	131 (24.9)
Tertiary (12+)	52 (9.9)
**Marital status**	
Married	294(55.8)
Single	103(19.5)
Divorced	70(13.3)
Widowed	60(11.4)
**Occupation**	
Unemployed	77(14.6)
Employed	287(54.5)
Privates	163(30.9)
**Living condition**	
Nuclear family	441 (83.7)
Extended family	9 (1.7)
Alone	77 (14.6)
**Family Size**	
< 4	314(59.6)
≥ 4	213(40.4)
**Residence**	
Urban	444(84.3)
Rural	83(15.7)
**Social Support**	
Poor	298(56.5)
Good	229(43.5)
**Perceived stigma**	
Yes	369(70%)
No	158(30%)
**Financial worries**	
Yes	360(68.3)
No	167(31.7)
**Family history of alcoholism**	
Yes	60(11.4)
No	467(88.6)
**History of Other Mental illness**	
Yes	5(0.9)
No	522(99.1)
**Family history mental illness**	
Yes	22(4.2)
No	505(95.8)
**History of Medical disorder**	
Yes	14(2.7)
No	513(97.3)
**Cigarette Smoking**	
Yes	30(5.7)
No	497(94.3)
**Khat chewing**	
Yes	44(8.3)
No	483(91.7)
**Missing ART drugs**	
Yes	50(9.5)
No	477(90.5)
**One or more substance use**	
Yes	385(73.1)
No	142(26.9)

### Prevalence of alcohol use disorder

**Fig 1:**
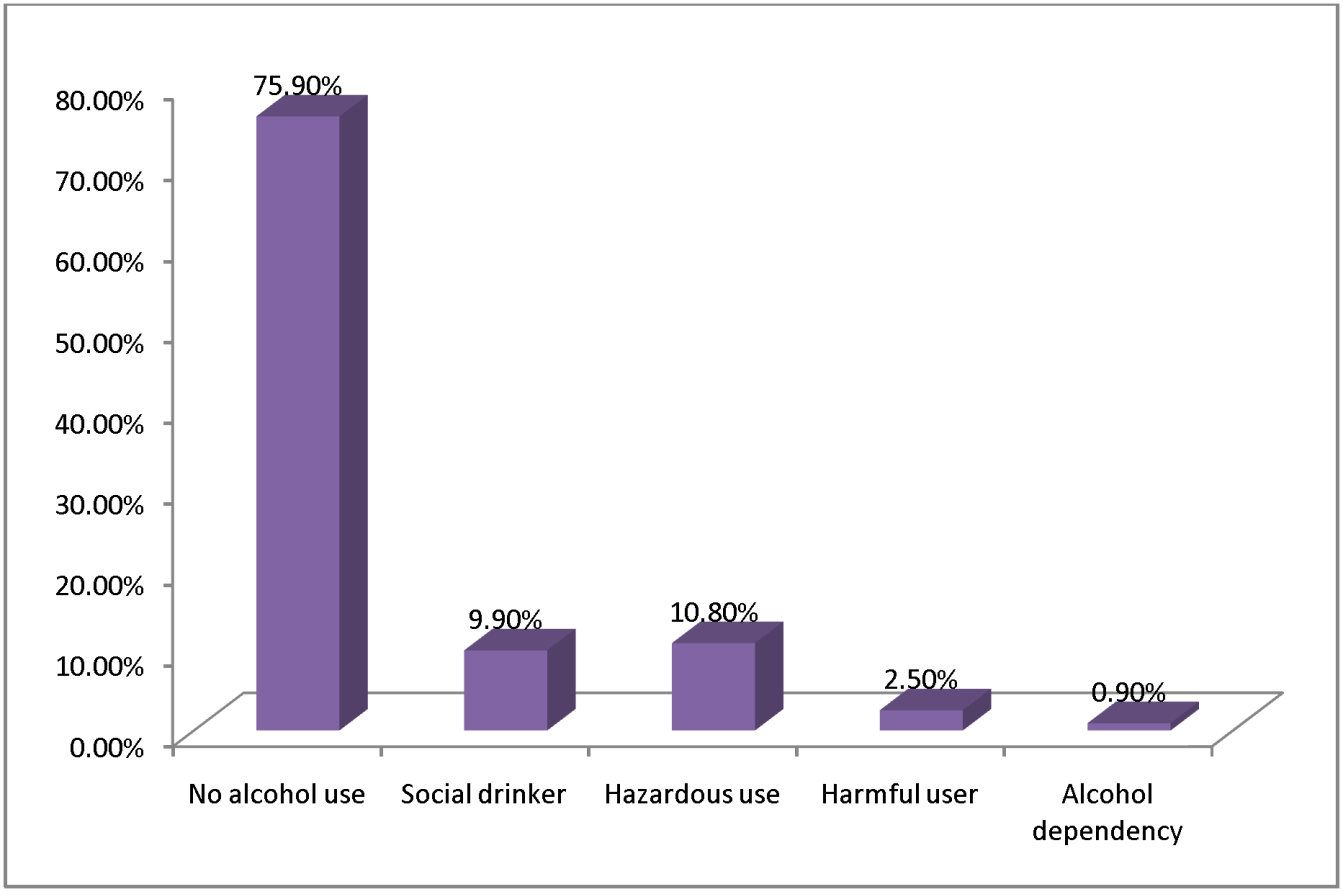
AUD and non AUD among HIV patients in ART clinlc at Bishoftu General Hospital 2015.

The prevalence of alcohol use disorder was found to be 14.2%. The prevalence of hazardous drinkers, harmful alcohol users, and alcohol dependents were computed to be 10.8%, 2.5%, and 0.9%, respectively. Most of the respondents, 400 (75.9%), were non-alcohol users and 9.9% were social drinkers. (Fig 1)

Easy availability of alcohol and using alcohol as a source of happiness were the main environmental and psychological factors for the initiation of alcohol use disorder among alcohol users in the study population which accounts for 14.6% and 4% of the cases respectively. The other factors for the initiation of alcohol use disorder reported by the participants were peer pressure, parental modeling, long standing life stressors, absence of support providers and long standing marital disharmony. (Table 2)

**Table 2:** Environmental and psychological factors for initiation of alcohol use disorders among people living with HIV and attending services at Bishoftu General Hospital ART Clinic 2015.

Reasons	n (%)
Easy availability of alcohol	**77(14.6)**
Like the way alcohol makes feel happy	**21(4)**
Peer pressure to drink	**18(3.4)**
Parental modeling	**9(1.4)**
Long standing life stressors	**8(1.5)**
Lack of social support.	**7(1.3)**
Long standing marital disharmony	**6(1.1)**
Drinking to forget financial difficulties	**2(0.4)**

In the bivariate analysis, sex, residence, wealth index, cigarette smoking, khat chewing, family history of alcoholism, family history of mental illness, and missing ART drugs were significantly associated with alcohol use disorder. (Table 3)

**Table 3:** Bivariate & multivariate logistic regression: Factors independently associated with AUD among people living with HIV attending services at Bishoftu General Hospital ART Clinic 2015 (n = 527).

Variables	AUD		COR	95% CI	AOR	95% CI
	Yes	No				
Age						
18–24	**2**	**35**		**		**
25–34	**30**	**169**	**3.10**	**(0.70, 13.60)**	**2.98**	**(0.51, 17.18)**
35–44	**25**	**162**	**2.70**	**(0.61, 11.93)**	**1.77**	**(0.29, 10.66)**
45–54	**16**	**60**	**4.66**	**(1.01, 21.50)**	**4.97**	**(0.74, 33.24)**
55 and above	**2**	**26**	**1.34**	**(0.17, 10.19**	**1.10**	**(0.09, 13.05)**
**Sex**						
Male	**49**	**165**	**3.27**	**(1.96, 5.47)**	**1.83**	**(0.89, 3.74)**
Female	**26**	**287**		**		**
**Residence**						
Urban	**56**	**388**	**0.48**	**(0.27, 0.87)**	**1.13**	**(0.42, 2.98)**
Rural	**19**	**64**		**		**
**Marital status**						
Married	**46**	**248**		**		**
Single	**17**	**86**	**1.06**	**(0.58, 1.95)**	**1.15**	**(0.50, 2.67)**
Divorced	**7**	**63**	**0.59**	**(0.25, 1.39)**	**0.43**	**(0.14, 1.27)**
Widowed	**5**	**55**	**0.49**	**(0.18, 1.29)**	**0.49**	**(0.15, 1.59)**
**Religion**						
Muslim	**3**	**13**		**		**
Orthodox	**68**	**351**	**0.84**	**(0.23, 3.02)**	**1.51**	**(0.18, 12.38)**
Protestant	**4**	**85**	**0.20**	**(0.04, 1.01)**	**0.48**	**(0.04, 5.02)**
**Ethnicity**						
Oromo	**43**	**231**		**		**
Amhara	**18**	**166**	**0.58**	**(0.32, 1.04)**	**1.63**	**(0.79, 3.41)**
Gurage	**7**	**25**	**1.50**	**(0.61, 3.69)**	**2.33**	**(0.43, 6.17)**
Tigray	**7**	**22**	**1.70**	**(0.68, 4.24)**	<**0.001**	**(0.61, 8.89)**
**Wealth index**						
Poorest	**8**	**97**		**		**
Poor	**17**	**89**	**0.26**	**(0.11, 0.61)**	**1.99**	**(0.64, 6.12)**
Intermediate	**13**	**92**	**0.61**	**(0.30, 1.21)**	**1.58**	**(0.47, 5.22)**
Rich	**12**	**94**	**0.45**	**(0.21, 0.94)**	**2.01**	**(0.58, 6.87)**
Richest	**25**	**80**	**0.40**	**(0.19, 0.86)**	**4.43**	**(1.30, 15.15)**
**Education**						
Illiterate	**19**	**77**	**2.96**	**(0.95, 9.23)**	**8.54**	**(1.70, 42.99)**
Primary	**32**	**216**	**1.77**	**(0.60, 5.26)**	**3.70**	**(0.85, 16.16)**
Secondary	**20**	**111**	**2.16**	**(0.70, 6.66)**	**2.98**	**(0.69, 12.90)**
Tertiary	**4**	**48**		**		**
**Occupation**						
Unemployed	**4**	**72**	**0.28**	**(0.10, 0.82)**	**0.55**	**(0.14, 2.14)**
Employed	**21**	**121**	**0.99**	**(0.51, 1.56)**	**1.79**	**(0.82, 3.87)**
Privates	**50**	**259**		**		**
**Social support**						
Poor	**35**	**263**	**0.62**	**(0.38, 1.02)**	**0.50**	**(0.26, 0.95)**
Good	**40**	**189**		**		**
**Smoking cigarette**						
Yes	**19**	**11**	**13.60**	**(6.15, 30.05)**	**1.49**	**(1.40, 2.13)**
No	**56**	**441**		**		**
**Chewing Khat**						
Yes	**24**	**20**	**10.16**	**(5.25, 19.67)**	**5.11**	**(1.60, 6.33)**
No	**51**	**432**		**		**
**History of Medical diseas**es						
Yes	**4**	**10**	**2.49**	**(0.76, 8.15)**	**2.19**	**(0.42, 11.24)**
No	**71**	**442**		**		**
**Family history of Alcoholism**						
Yes	**20**	**40**	**3.74**	**(2.04, 6.86)**	**3.58**	**(1.51, 8.47)**
No	**55**	**412**	**0.13**	**		**
**Family history of Psychiatry problem**						
Yes	**7**	**15**	**2.99**	**(1.18, 7.62)**	**2.04**	**(0.53, 7.86)**
No	**68**	**437**		**		**
**Missing ART drugs**						
Yes	**19**	**31**	**4.60**	**(2.44, 8.69)**	**3.04**	**(1.30, 7.13)**
No	**56**	**421**	**0.13**	**		**

**Reference catagory

The multivariate analysis was used to identify factors that were predictive of alcohol use disorder. Educational status, smoking cigarettes, chewing khat, family history of alcohol use, and missing ART drugs were independently associated with Alcohol use disorder. (Table 3)

## Discussion

The study reveals that the prevalence of Alcohol Use Disorder (AUD) was 14.2 which is higher that of the community and facility based studies in some African countries. On the other hand, the current finding is in line with the empirical knowledge and research outputs of the field. [1, 3, 5]. Therefore, this study gives additional evidence for designing interventions for alcohol use disorder for HIV infected patients.

An overview of previously done global studies on the prevalence of AUD among people living with HIV/AIDS provides an abundance of statistics that range from 1.4% in Uganda, 22.5% in South Africa, 23% in US America, 34.8% in South Africa, 39.4% in Nigeria, and 49.5% in Brazil[20–24]. AUD prevalence detected in this study was lower than that of South Africa, US America, Nigeria and Brazil. The possible explanation for this might be socio-economic, cultural, and contextual differences. The sample size variations and instruments used to assess AUD differences were also the possible reasons that make the dissimilarity at both ends [20–24].

Khat chewing is positively associated with AUD. HIV infected patients who chew khat had 5 times more risk for AUD as compared to non khat chewers. This finding is in line with that of a study done in Jimma[25]. Therefore, this study gives additional evidence for planning appropriate intervention in khat chewing HIV infected patients.

Cigarette smoking in HIV infected patients was positively associated with AUD in which those who smoke had 1.49 times greater risk than non smokers. This finding is in agreement with those of multi country studies [17, 25–27]. The possible explanation for this might be that smoking cigarettes and drinking alcohol are interrelated.

Social support is significantly associated with AUD. HIV infected patients with poor social support had 50% more risk for AUD as compared to those who had good social support. This is in agreement with the longitudinal follow-up study done in southwest Ethiopia[28]. Therefore, the presence of good social support will overcome the use of alcohol and to have good drug adherence.

Family history of alcoholism was found to be significantly associated with AUD. HIV infected patients who had family history of alcoholism had 3.58 times more risk of AUD as compared to those who had no family history of alcoholism. This finding is in line with that of a study done in Jimma[17]. This reveals that family history of alcoholism has increased the risk of drinking alcohol.

Missing ART drug was found to be associated with AUD. Patients who missed their ART drug had 3 times more risk of AUD as compared to those who didn’t miss their ART drug. The relationship between missing ART and AUD is complex and bidirectional. This could be explained in such a way that excessive use of alcohol might result in cognitive impairment and that affects ART drug adherence. On the other hand, missing ART drugs increases stress level and exposure to opportunistic infections. Hence, people might use alcohol as a coping strategy for their stress.

In contrast to other studies, age and sex[17, 25] were not found to be significantly associated with AUD.

The findings of this study should be interpreted with some limitation in mind. As this is a cross-sectional study, causal inference cannot be made between AUD and independent variables. Administering the questionnaire through a face to face interview might have resulted in a socially desirable response. Therefore, there might be a social desirability bias.

## Conclusion

The prevalence of Alcohol Use Disorder was high. Educational status, social support, cigarette smoking, khat chewing, family history of alcohol use, and missing ART drugs were independently associated with alcohol use disorder. Providing Health education about alcohol use and proper screening of alcohol use disorder among patients with HIV/AIDS are crucial. Strengthening the referral linkage with psychiatric units decreases the burden of the problem.

## Ethical considerations

Ethical clearance was obtained from Institution Review Board (IRB) of the University of Gondar. Then, the official permission and letter of collaboration obtained from the University of Gondar and Amanuael Mental Specialized Health Hospital (AMSH) were submitted to Bishoftu General Hospital. An official letter of permission was obtained from the Administration of Bishoftu General Hospital. The purpose of the study was well explained to the study participants and their informed consent was obtained. Confidentiality was maintained at all levels of the study by avoiding use of names and other identifiers. Participants’ involvement in the study was voluntary; participants who were unwilling to participate in the study and those who wished to quit were informed to do so without any restriction. Patients with AUD were referred to a mental health professional.

## Abbreviations

AOR: Adjusted odds ratio
ART: Antiretroviral therapy
AUD: Alcohol use disorder
AUDIT: Alcohol use disorder Identification test
HIV: Human Immunodeficiency Virus
IRB: institutional review board
SPSS: statistical package for social sciences
